# Cyclic-AMP Mediated Regulation of *ABCB* mRNA Expression in Mussel Haemocytes

**DOI:** 10.1371/journal.pone.0061634

**Published:** 2013-04-12

**Authors:** Silvia Franzellitti, Elena Fabbri

**Affiliations:** 1 Interdepartment Centre for Environmental Science Research, University of Bologna, Ravenna, Italy; 2 Department of Biological, Geological, and Environmental Sciences, University of Bologna, Bologna, Italy; Temple University, United States of America

## Abstract

**Background:**

The multixenobiotic resistance system (MXR) allows aquatic organisms to cope with their habitat despite high pollution levels by over-expressing membrane and intracellular transporters, including the P-glycoprotein (Pgp). In mammals transcription of the ABCB1 gene encoding Pgp is under cAMP/PKA-mediated regulation; whether this is true in mollusks is not fully clarified.

**Methodology/Principal Findings:**

cAMP/PKA regulation and *ABCB* mRNA expression were assessed in haemocytes from Mediterranean mussels (*Mytilus galloprovincialis*) exposed *in vivo* for 1 week to 0.3 ng/L fluoxetine (FX) alone or in combination with 0.3 ng/L propranolol (PROP). FX significantly decreased cAMP levels and PKA activity, and induced *ABCB* mRNA down-regulation. FX effects were abolished in the presence of PROP. *In vitro* experiments using haemocytes treated with physiological agonists (noradrenaline and serotonin) and pharmacological modulators (PROP, forskolin, dbcAMP, and H89) of the cAMP/PKA system were performed to obtain clear evidence about the involvement of the signaling pathway in the transcriptional regulation of *ABCB*. Serotonin (5-HT) decreased cAMP levels, PKA activity and *ABCB* mRNA expression but increased the mRNA levels for a putative 5-HT_1_ receptor. Interestingly, *5-HT_1_* was also over-expressed after *in vivo* exposures to FX. 5-HT effects were counteracted by PROP. Forskolin and dbcAMP increased PKA activity as well as *ABCB* mRNA expression; the latter effect was abolished in the presence of the PKA inhibitor H89.

**Conclusions:**

This study provides the first direct evidence for the cAMP/PKA-mediated regulation of *ABCB* transcription in mussels.

## Introduction

Environmental physiologists often link the endogenous processes of organisms with exogenous stimuli affecting them in order to understand the impact of pollution on the broader distributions of populations and species. However, only a few molecular mechanisms driving stress tolerance and adaptation in aquatic organisms have been elucidated to date; these include pathways of response to thermal [1] or metal [2] stress.

Marine mussels are intertidal organisms living in rapidly fluctuating habitats where protection from natural and anthropogenic stressors (i.e. temperature or salinity daily and/or seasonal variations, metals, PAHs and other contaminants) is essential for survival. Although the responses are often behavioral or metabolic, a powerful mechanism employed by these organisms to cope with environmental challenges is the regulation of genes and proteins related to cytoprotection. These include the multixenobiotic resistance system (MXR), which prevents the cellular accumulation of potentially harmful xenobiotics by active export from the cell of parental or metabolized forms of the compounds. In mussels the MXR response was investigated to infer stress tolerance in animals inhabiting contaminated environments [3], [4], and experimental evidence indicated that this system provides a powerful adaptive advantage for marine bivalves to cope with environmental challenges [5].

Several proteins are involved in the MXR system, and the most studied in an environmental context is the P-glycoprotein (Pgp) [5]. Pgp induction in mussels has been reported in response to a wide range of chemical and physical stressors, including metals, pesticides, as well as temperature or salinity variations [3], [5], suggesting that this transporter may be part of a general and broad-spectrum cellular stress response.

Evidence indicates that in mammals transcriptional regulation of the ABCB1 gene encoding Pgp is mediated through the phosphorylation activity of the cAMP-dependent protein kinase (PKA). This regulatory pathway was well characterized in tumor cells displaying a constitutive *ABCB1* over-expression that acquired chemoresistance [6]. Conversely, the involvement of cAMP has only been hypothesized in mollusks [7]–[9].

In the present work, *in vivo* and *in vitro* experiments were performed to investigate the modulation of the cAMP/PKA pathway and the putative downstream effects on mRNA expression of an ABCB gene in Mediterranean mussels (*Mytilus galloprovincialis*).

*In vivo* experiments were carried out exposing mussels to fluoxetine (FX) alone or in combination with propranolol (PROP). FX has recently received considerable attention in the framework of risk assessment investigations with emerging contaminants due to its frequent detection in aquatic environments [10]; moreover, it is recognized as one of the human pharmaceuticals with the highest acute toxicity toward non target organisms [10]. FX is the active ingredient of the antidepressant Prozac®, the most widely prescribed psychoactive drug in the market, acting as selective serotonin reuptake inhibitor (SSRI) in the treatment of depression and other mood disorders by increasing the serotonin levels in neuron synaptic space [11]–[13]. Serotonin (5-hydroxytryptamine, 5-HT) is involved in hormonal and neuronal mechanisms and plays a key role in regulating food intake, metabolism and reproductive success in invertebrates [14], [15]. By interfering with serotoninergic regulation, FX has, therefore, the potential to impair relevant physiological functions in invertebrates.

PROP is a β adrenergic receptor antagonist used in human therapies to counteract cardiovascular pathologies [16], but it can also act as a 5-HT receptor antagonist [14]. PROP is widely detected in aquatic environments [17]–[19]. The drug was recently reported to bioconcentrate up to about 360 µg/g w.w. in mussel tissues [20], and also to affect cAMP signaling and *ABCB* mRNA expression [8].

To specifically address different steps of the pathway potentially leading to regulation of ABCB mRNA expression, *in vitro* experiments using several physiological agonists and pharmacological modulators of cAMP/PKA signaling were carried out on isolated haemocytes. Besides the advantages provided by their employment as a cell model for both *in vivo* and *in vitro* investigations of ABCB gene regulation in a nonconventional model species as the marine mussel [21], haemocytes represent an attractive model for the purposes of this study. These cells are known to have a complex cell signaling network that allows them to modulate their own functions [22]. These signaling pathways show high homology with those of vertebrates [23], [24]; however, their physiological roles in mussel haemocytes need further study.

## Results

### Effects of FX, PROP or the mixture FX+PROP on cAMP-related parameters and *ABCB* mRNA expression under *in vivo* exposures

Mussels were exposed *in vivo* to fluoxetine (FX), a selective serotonin reuptake inhibitor, to propranolol (PROP), a β-adrenergic receptor blocker, or to their mixture (FX+PROP). The tested concentration for both compounds (0.3 ng/L) falls within the low environmental range reported in surface and coastal waters [13], [25].

**Figure 1:**
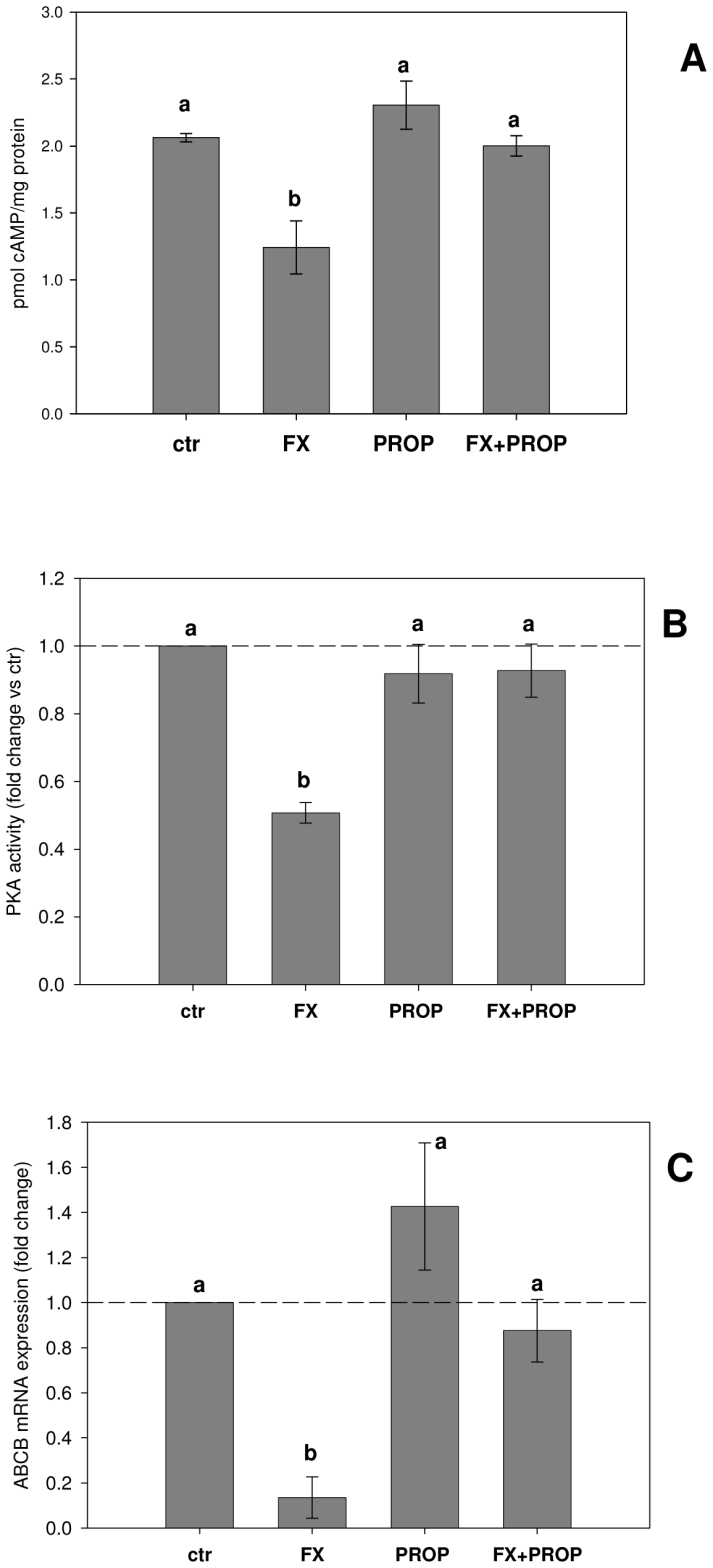
The pharmaceutical FX alters cell signaling endpoints and *ABCB* mRNA expression in mussel haemocytes under *in vivo* exposures. (A) cAMP content (B) relative PKA activities (C) relative *ABCB* mRNA expression. All biological endpoints were assessed in haemocytes collected from mussels exposed for 7 days to 0.3 ng/L FX 0.3 ng/L PROP, or to the mixture FX+PROP (0.3 ng/L+0.3 ng/L). A group of control (water) exposed mussels were included in the analysis. Data represent mean ± SEM (N = 5). Different superscripts letters indicate statistically significant differences (p<0.05, one-way ANOVA followed by Bonferroni's test). Basal PKA activity = 0.88±0.07 nmol/min/mg protein.

Haemocytes collected from mussels exposed to FX showed significantly decreased cAMP levels and PKA activity above control values, and also *ABCB* mRNA down-regulation (**Fig. 1**). Mussel exposure to the mixture FX+PROP did not induce significant variations of the biological endpoints with respect to control levels (**Fig. 1**).

### Expression of the mussel *ABCB* gene product is affected by serotoninergic but not by adrenergic agonists

**Figure 2:**
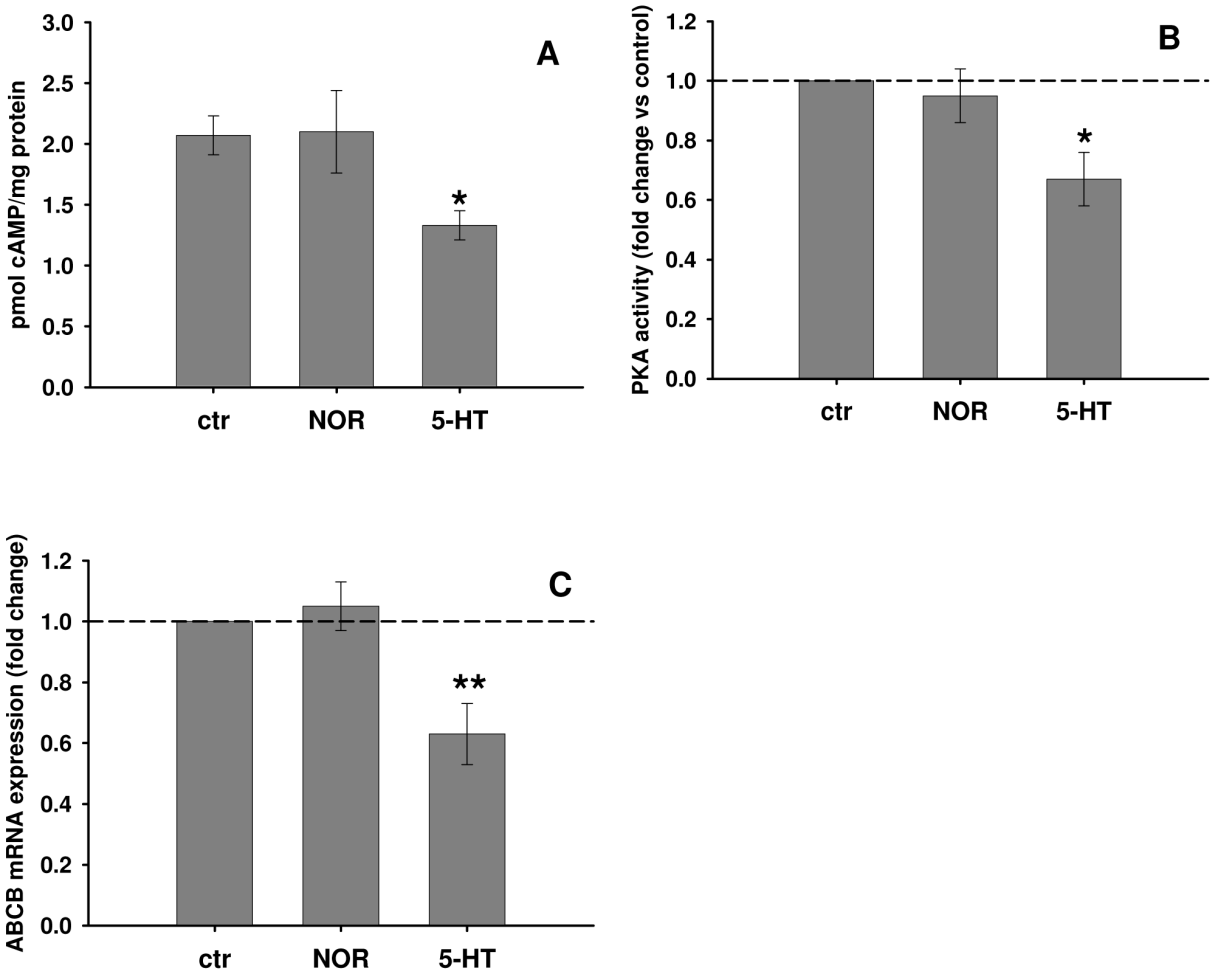
Effects of NOR and 5-HT on cAMP-related parameters and *ABCB* mRNA expression in mussel haemocytes incubated *in vitro*. (A) cAMP content; (B) relative PKA activities; (C) relative *ABCB* mRNA expression. All biological endpoints were assessed after 1 h of exposure to 1 µM NOR or 5-HT. Data are derived from three independent experiments (mean ± SEM); *p<0.05 *vs* control. Basal PKA activity = 1.12±0.17 nmol/min/mg protein.

Haemocytes from control *M. galloprovincialis* were treated with either noradrenaline (NOR) or serotonin (5-HT) as selective agonists of adrenergic (AR) and 5-HT receptors, respectively. NOR did not increase either cAMP levels or PKA activities, whereas 5-HT addition significantly reduced both endpoints (**Fig. 2A,B**). Accordingly, *ABCB* mRNA expression was not affected by NOR while it was significantly down-regulated by 5-HT (**Fig. 2C**).

**Figure 3:**
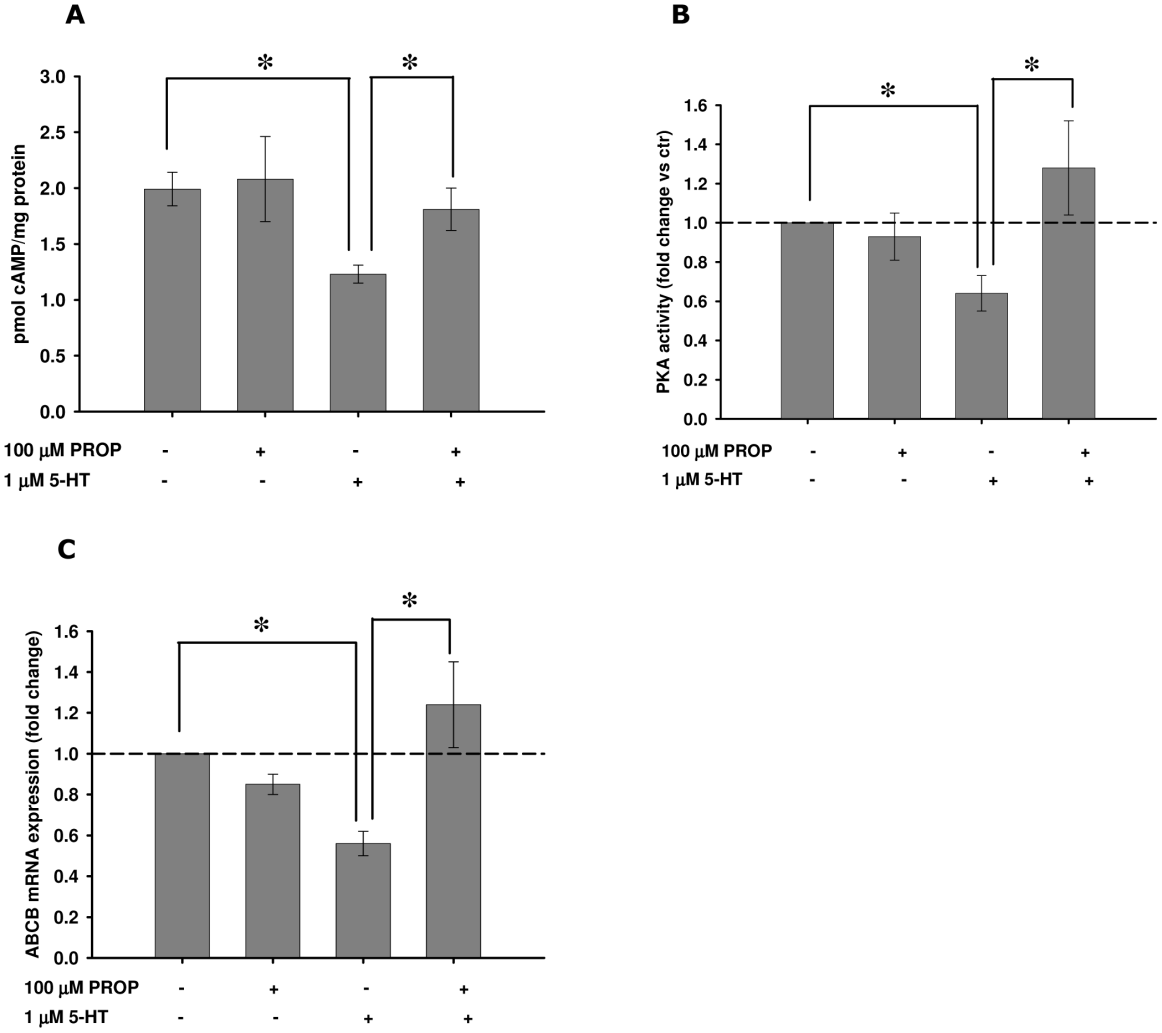
Effects of PROP pre-incubation on 5-HT modulated parameters in mussel haemocytes incubated *in vitro*. (A) cAMP content; (B) relative PKA activities; (C) relative *ABCB* mRNA expression; (D) relative *5-HT_1_* mRNA expression. All biological endpoints were assessed after 1 h of exposure to 1 µM 5-HT in the presence/absence of a 15-min pre-incubation with 100 µM PROP. Data are derived from three independent experiments (mean ± SEM); *p<0.05 between pairs of samples. Basal PKA activity  =  1.12±0.17 nmol/min/mg protein.

The effects of 5-HT on mussel haemocytes were further evaluated in the presence of PROP, used as a 5-HT_1_ receptor antagonist [26], [27]. PROP significantly blocked the reduction in cAMP levels and PKA activities and the down-regulation of the *ABCB* gene product triggered by 5-HT (**Fig. 3A,B,C**).

### Expression of a mussel *5-HT_1_* gene product is up-regulated by 5-HT

**Figure 4:**
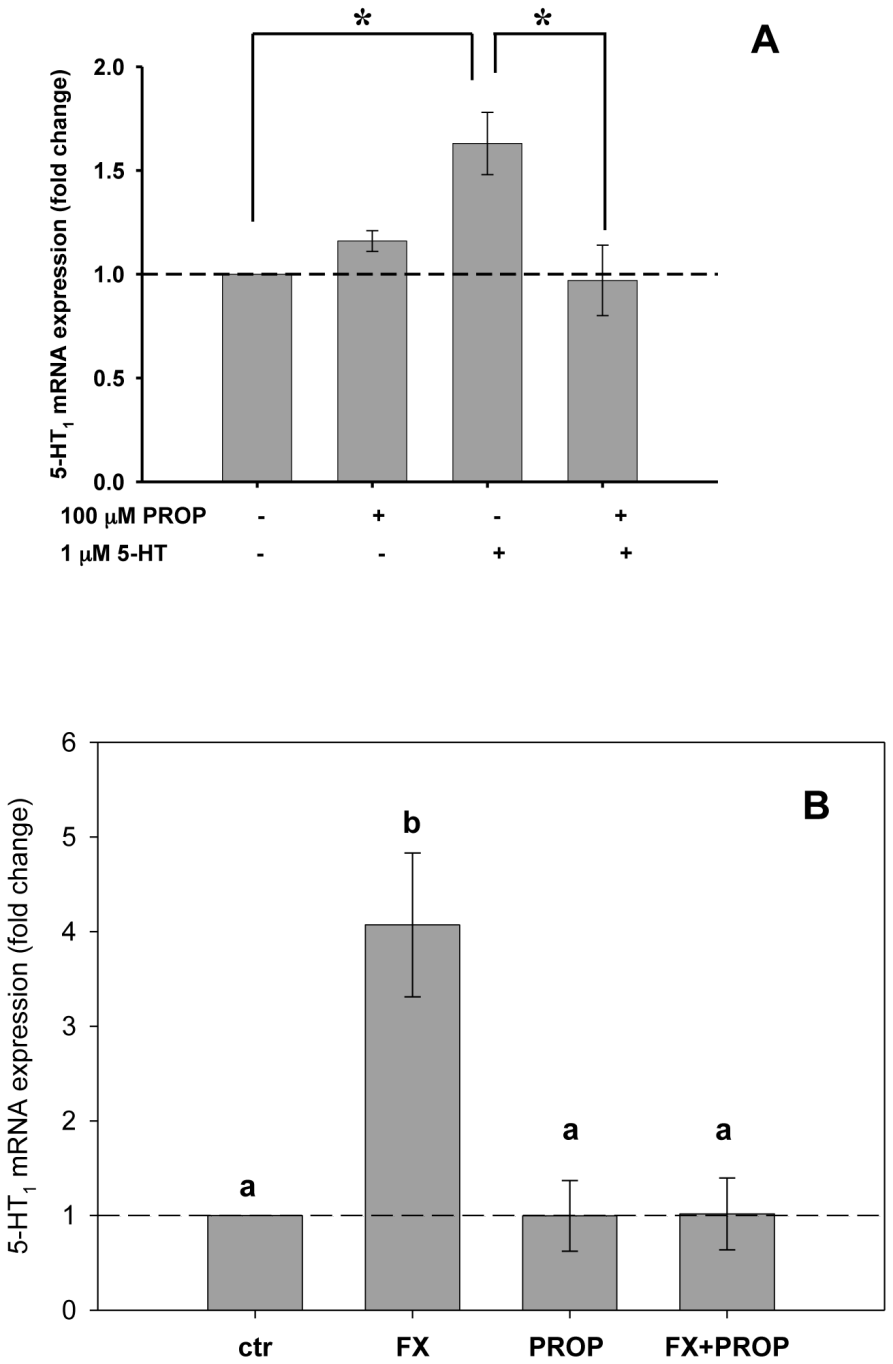
Relative mRNA levels of a 5-HT_1_ receptor are affected by 5-HT or FX exposure. (**A**) Relative *5-HT_1_ mRNA* expression assessed in haemocytes incubated *in vitro* for 1 h with 1 µM 5-HT in the presence/absence of a 15-min pre-incubation with 100 µM PROP. Data are derived from three independent experiments (mean ± SEM); *p<0.05 between pairs of samples. (**B**) Relative *5-HT_1_ mRNA* expression evaluated in haemocytes of mussels exposed *in vivo* for 7 days to FX (0.3 ng/L), PROP (0.3 ng/L) or to the mixture FX+PROP (0.3 ng/L+0.3 ng/L). A group of control (water) exposed mussels were included in the analysis. Data are reported as mean ± SEM. Different superscripts letters indicate statistically significant differences (p<0.05, N = 5).

As shown in **Fig. 4A**, a 1-hour incubation with 5-HT *in vitro* also induced a significant up-regulation of a transcript encoding a putative mussel 5-HT_1_ receptor (*5-HT_1_*) in haemocytes, while cell pre-treated with PROP significantly blocked the 5-HT effect on its receptor. In agreement, also haemocytes from mussels exposed *in vivo* to FX showed a significant up-regulation the *5-HT_1_* transcript (**Fig. 4B**). This effect was not observed in mussels exposed to the mixture FX+PROP (**Fig. 4B**).

### The *ABCB* gene product is over-expressed following FSK-activated cAMP/PKA signaling

**Figure 5:**
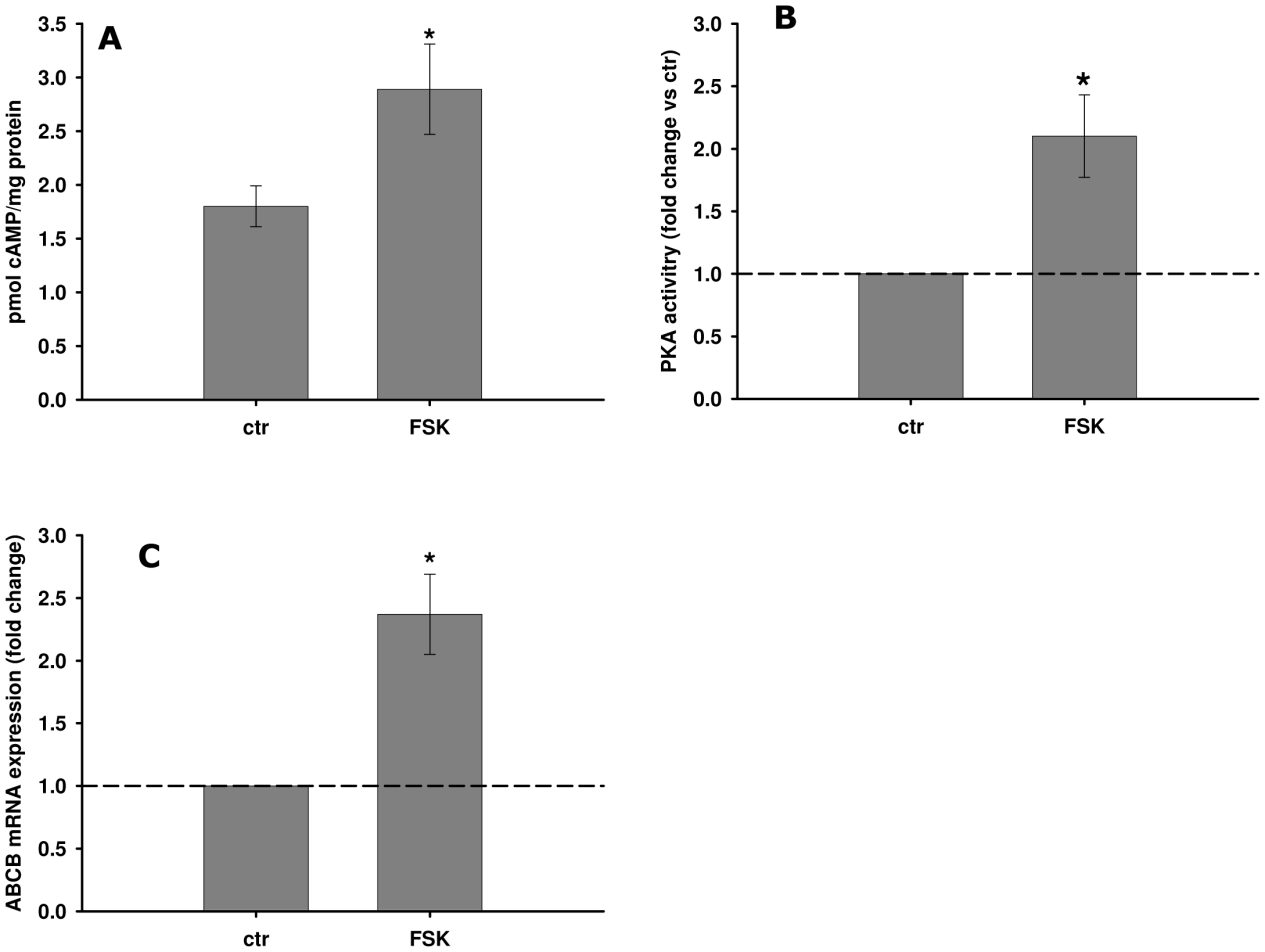
Effects of FSK on cAMP-related parameters and *ABCB* mRNA expression in mussel haemocytes incubated *in vitro*. (A) cAMP content; (B) relative PKA activities; (C) relative *ABCB* mRNA expression. All biological endpoints were assessed after 4 h of exposure to 20 µM FSK. Data are derived from three independent experiments (mean ± SEM); *p<0.05 *vs* control. Basal PKA activity = 1.12±0.17 nmol/min/mg protein.

The adenylyl cyclase (AC) activator forskolin (FSK) was used to investigate the effect of cAMP/PKA stimulation on *ABCB* mRNA expression. Indeed, FSK induced significant increases in cAMP levels and PKA activities in mussel haemocytes (**Fig. 5A,B**), which was correlated with a significant *ABCB* transcript up-regulation (**Fig. 5C**).

### Expression of the *ABCB* gene product follows induction and inhibition of PKA activity

**Figure 6:**
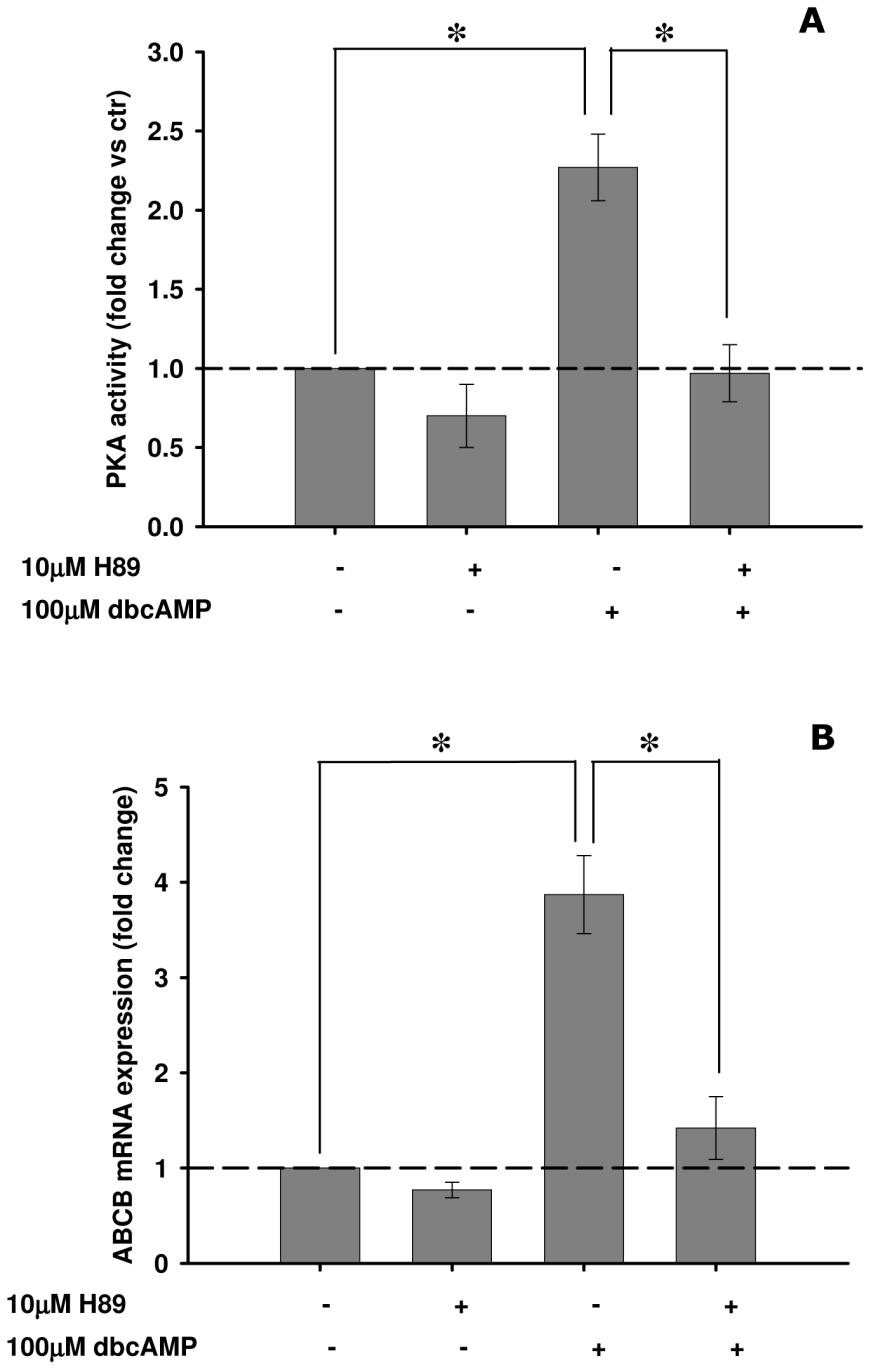
Effects of H89 pre-incubation on dbcAMP induced stimulation of PKA activity and *ABCB* mRNA expression in mussel haemocytes incubated *in vitro*. (A) relative PKA activities; (B) relative *ABCB* mRNA expression. Both biological endpoints were assessed after 4 h of exposure to 100 µM dbcAMP in the presence/absence of a 30-min pre-incubation with 10 µM H89. Data are derived from three independent experiments (mean±SEM); *p<0.05 between pairs of samples. Basal PKA activity = 1.12±0.17 nmol/min/mg protein.

Haemocytes were exposed for 4 h to the PKA activator dbcAMP in the presence/absence of H89, a selective PKA inhibitor (**Fig. 6**). As predicted, dbcAMP significantly increased PKA activities, while no significant effects were detected in the presence of H89 (**Fig. 6A**). *ABCB* mRNA levels were significantly increased by haemocyte dbcAMP treatment, while cells treated with dbcAMP in the presence of H89 had activities not significantly different from the control (**Fig. 6B**).

### Sequence analysis of the 5′-untranslated region of mussel ABCB genes

**Figure 7:**
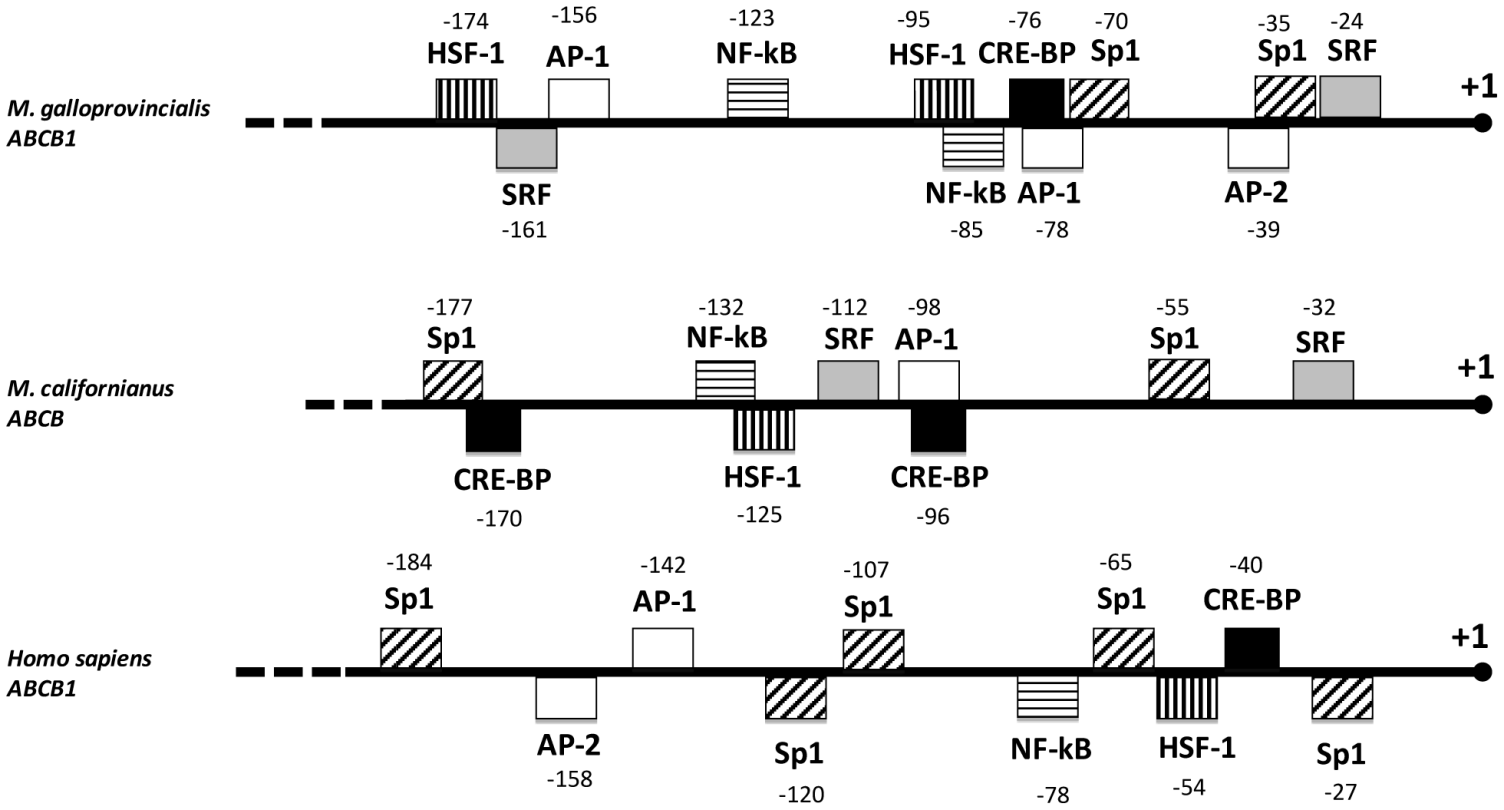
Untranslated 5′ regulatory region of ABCB genes from the mussels *M. galloprovincialis and M. californianus* showing promoter elements. Numbering is relative to the transcription initiation site. Gene Bank Accession Numbers are FM999809 (*M. galloprovincialis* ABCB1) and EF52141 (*M. californianus* ABCB). Sequence of the human ABCB1 promoter in the same region is reported for comparison (GenBank Ac. Numb. NM000927). Furthermore, a more complete sequence analysis of the human ABCB1 promoter is reported by Scotto and Johnson [6] and Rohlff and Glazer [28].

The computational analysis of the 5′-untranlasted regions of ABCB genes from two mytilid species M. galloproviancialis and M. californianus revealed the presence of several putative PKA-related regulatory elements within the ABCB promoter region (**Fig. 7**). Amongst these, putative binding sites for the PKA-dependent CRE-BP transcription factor were found in both M. galloprovincialis (−76) and M. californianus (−96 and −170) ABCB promoter regions; binding sites for this transcription factor are also present within the human ABCB1 gene promoter ([28]; **Fig. 7**).

Regulatory elements binding the AP-1 and AP-2 transcription factors, which occur in the human ABCB1 promoter and are regulated by PKA in a manner similar to CREB ([28] and reference therein), were also found in the 5′untranslated regions of both mussel ABCB gene sequences analysed (**Fig. 7**). Moreover, binding sites for the Sp1 transcription factor were also found at position −35 (M. galloprovincialis), and at positions −55 and −177 (M. californianus) (**Fig. 7**). Sp1 is a PKA-regulated nuclear transcription factor that binds to TATA-less promoters, including ABCB gene promoters, and it is primarily involved in constitutive rather than stress-induced gene regulation [6], [29]. Binding sites for the HSF transcription factor were found both in M. galloprovincialis (−95, −174) and M. californianus (−125) ABCB promoter regions (**Fig. 7**). Finally, binding sites for SRF and NF-kB transcription factors were also found in ABCB promoter regions from both mussel species (**Fig. 7**), consistent with reports for the human ABCB gene promoter [6].

## Discussion

Data reported in this study showed that the impairment of transduction pathways caused by low concentrations of pollutants have the potential to affect the ability of animals to elaborate strategies of defense or adaptation. Indeed, exposure of mussels to low environmental range concentrations of the pharmaceutical fluoxetine affected cell signaling mediators such as cAMP steady-state levels, PKA activity, and mRNA levels for a 5-HT_1_ receptor in haemocytes, and also altered mRNA levels of an ABCB gene encoding the membrane transporter P-glycoprotein, which displays a peculiar function in cellular defense machinery [5].

The mode of action of FX as a selective serotonin reuptake inhibitor (SSRI) is to increase serotoninergic neurotransmission at mammalian synapses by blocking removal of 5-HT by reuptake transporters ([28] and reference therein). As such, FX mimics the action of a continuous exposure to increased extracellular levels of 5-HT ([30] and reference therein). As discussed in detail below, the physiological functions of serotonin are mediated by multiple membrane receptors coupled to the stimulation of phospholipase C (PLC) or to the induction/inhibition of adenylyl cyclase (AC) in mammals, and several lines of evidence suggest a similar scenario also in bivalves, although the pharmacological classification of 5-HT receptors in these organisms is incomplete and yet limited to what reported by Tierney [14]. The reduction of cAMP levels and PKA activities below control values observed in this study are, therefore, consistent with the inhibition of AC activity by a putative increase of 5-HT extracellular levels induced by FX. As a further support to this hypothesis, FX effects were completely abolished when mussels were exposed to FX in the presence of PROP. Indeed, although PROP is a prototypical β-AR antagonist used in human therapies to counteract cardiovascular pathologies [16], it is also an effective antagonist for type 1 5-HT receptors (5-HT_1_), which are coupled to AC inhibition [14], [26], [27]. Therefore, both compounds can affect the same molecular target indirectly (FX) by increasing the availability of the agonist molecules in the extracellular medium, directly (PROP) by binding to the receptor and avoiding its occupation by the agonist.

Data from *in vivo* exposures also showed that *ABCB* mRNA expression profile is consistent with variations of cAMP-related parameters. Considering the specific effect displayed by FX and PROP on cAMP signaling, these data provided further hints about a cAMP/PKA involvement in the transcriptional regulation of the mussel ABCB gene encoding the P-glycoprotein. Besides present results, other in vivo investigations provided some indirect evidence towards this connection Indeed, mussels exposed to environmental concentrations of carbamazepine, whose therapeutic mode of action in humans is at least in part linked to the cAMP/PKA signaling pathway, showed significant variations of intracellular cAMP levels and PKA activities, changes which were correlated with the regulation of *ABCB* mRNA expression in different tissues [7], [8]. Further environmental pollutants, including metals or algal products, are reported to alter both cAMP levels and MXR-related mRNA expression in mussels [9], [31].

**Figure 8:**
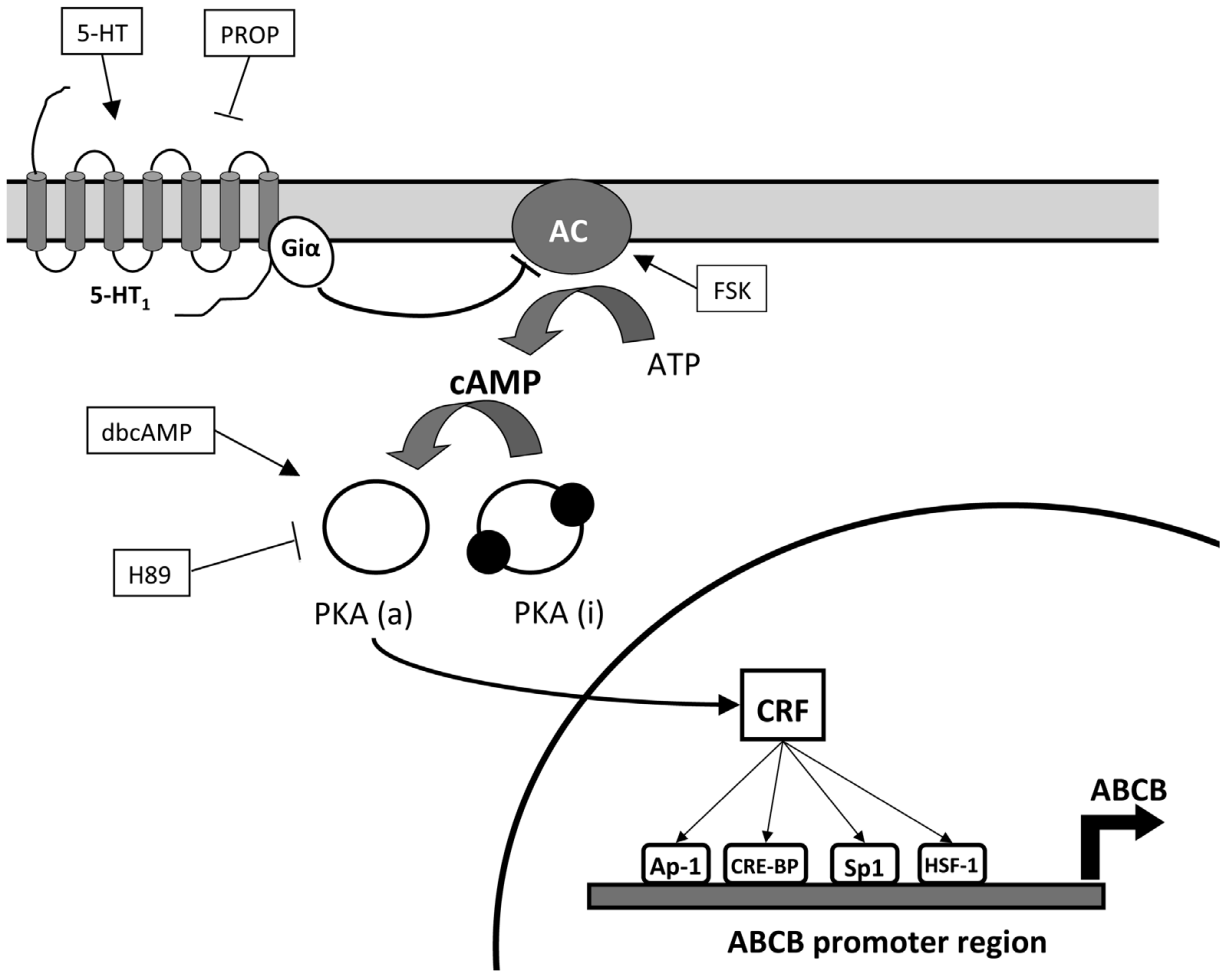
Schematic representation of the cAMP-dependent pathway leading to *ABCB* transcriptional regulation in mussel haemocytes. 5-HT_1_: type 1 serotonin receptor; 5-HT: serotonin; PROP: propranolol; Gi: inhibitory G-protein; AC: adenylyl cyclase; FSK: forskolin; cAMP: cyclic-AMP; PKA(i): inactivated cAMP-dependent protein kinase (PKA); PKA(a): activated PKA (catalytic subunit); dbcAMP: dybutyril cAMP; H89: N-[2-((p-Bromocinnamyl)amino)ethyl]-5-isoquinolinesulfonamide dihydrochloride; CRF: cAMP-responsive factors.

To provide clear demonstration about the involvement of cAMP and PKA in the modulation of the mussel MXR system, and more specifically in the transcriptional regulation of the ABCB gene, *in vitro* investigations using haemocytes as a cell model were performed. To our knowledge, the data represent the first direct evidence that the regulatory framework, well documented in mammals, is evolutionary-conserved in bivalve mollusks (**Fig. 8**).

Haemocytes were treated with noradrenaline (NOR) or 5-HT as the physiological agonists known to modulate cAMP levels through adrenergic and serotoninergic receptors, respectively, and both are reported as the main neuromodulators in mussels [15].

Data on the expression and distribution of adrenergic receptors in bivalves is pending, although indirect evidence suggest the occurrence of α-AR and β-AR in all bivalve tissues investigated to date (reviewed in [15]). NOR is the main catecholamine found in bivalves [32]–[35], and NOR levels readily increase in response to different stressors commonly faced by bivalves in their natural habitat [34]. NOR concentrations used in this study (1 µM) are 10-time higher than that reported in circulating fluids under stress conditions [34]. Nevertheless, NOR was ineffective on haemocyte cAMP signaling and *ABCB* mRNA expression, although Lacoste et al. [34], [35] showed that NOR concentrations ranging from 0.1 to 10 µM decreased phagocytosis and increase hsp70 promoter activity in oyster haemocytes probably through a β- and a α-AR signaling pathway, respectively. It must be stressed that the effect of NOR on the signaling pathway was not assessed in the above studies. According to our results, AR receptors coupled to cAMP regulation are either absent or not responsive to NOR in mussel haemocytes. Conversely, 5-HT significantly decreased cAMP levels in haemocytes.

Mammalian 5-HT receptors are classified into seven distinct classes (5-HT_1_, 5-HT_2_, etc.), subdivided in subclasses and coupled to an enormous array of behavioral and biochemical effects [36]. In particular, mammalian 5-HT_1_ and 5-HT_7_ are coupled to the inhibition and activation of AC, respectively, while 5-HT_2_ receptors are coupled to the stimulation of PLC, leading to Ca^2+^ increases and PKC activation. Phylogenetic studies reported that the main subfamilies of 5-HT receptors diverged early in evolution to form three major subclasses: 5-HT1 (which includes 5-HT5 and 5-HT7), 5-HT2, and 5-HT6 receptor subclasses [37]. This divergence occurred before the branching of vertebrates from invertebrates, and thus finding members of these major subclasses in invertebrate species would be expected [38]. Further division within the 5-HT1 and 5-HT2 subfamilies seemed to have occurred after the branching of vertebrates and invertebrates, and these subtypes have evolved independently within vertebrates and invertebrates [14], [39]. Therefore, it is not surprising to find that pharmacological profiles of molluscan and mammalian 5-HT receptors are difficult to compare, considering the large phylogenetic distances involved [39]. Studies examining both functional and molecular characterization of 5-HT receptors in mollusks reported that 5-HT_1_ subtypes are reminiscent of an ancestor 5-HT gene that existed before the divergence of the 5-HT receptor subtypes in vertebrates, and kept characteristics of more than one receptor subtype [39], [40]. This particular receptor subtype seems negatively coupled to the cAMP pathway in invertebrates as well as in mammals [14], which is in line with the results from the present experiments. A partial sequence encoding a 5-HT receptor structurally homologous to the mammalian 5-HT_1_ sub-group was recently obtained from the gonads of another bivalve mollusk, Mytilus edulis [41], and found to be expressed also in digestive gland of *M. galloprovincialis* [42]. Gene expression analyses reported in this study and that of Ciacci et al. [43] observed the expression of this putative 5-HT_1_ receptor in mussel haemocytes. Moreover, mRNA levels for this receptor were increased in response to 5-HT stimulation, in agreement with a previous study in the brain of a shrimp by Tiu et al. [44]. These authors hypothesized that in the continuous presence of the agonist, a feedback regulatory mechanism for *5-HT_1_* mRNA expression in invertebrate cells may exist as new receptors may be needed to replace old receptors, or alternatively, new receptors are needed when all other receptors are occupied [44]. In agreement with the reported findings, both the *in vivo* effects of FX and the *in vitro* effects of 5-HT examined in this study lead to an increase of *5-HT_1_* mRNA levels.

Besides lowering cAMP levels, 5-HT also reduced PKA activities in mussel haemocytes, again in agreement with the occupation of 5-HT_1_ receptors. Concomitantly, the agonist reduced *ABCB* mRNA expression. Interestingly, the antagonist PROP prevented the up-regulation of *5*-*HT_1_* mRNA expression *in vitro* as well as *in vivo*, counteracting all the downstream effects induced by the agonist.

Pharmacological ligands known to increase cAMP levels and/or PKA activities increased also *ABCB* mRNA expression. These included FSK, which directly activates the enzyme adenylyl cyclase, and dbcAMP, a lipophilic analog of cAMP, which specifically activates PKA activities. The latter effect was blocked by haemocyte pre-incubation with H89, the selective inhibitor of PKA. These findings clearly indicate that the cAMP/PKA pathway exerts a significant control over *ABCB* transcription. Indeed, in mammalian cells transcriptional regulation of the ABCB1 gene requires the PKA-mediated activation of several transcription factors (TFs) that are phosphorylated by PKA after cAMP activation of the holoenzyme, and bind to consensus sequences in the promoter region of the mammalian ABCB1 gene [28], [45], [46]. PKA has been extensively studied in mussels, showing a high degree of structural and functional homology with its mammalian counterpart [47], [48]. In particular a PKA isoform (namely PKA_myt1_) homologous to the human type-I PKA was reported in mussel haemocytes [48]; this protein isoform corresponds to the one involved in ABCB1 transcriptional regulation in mammals [28].

Furthermore, sequence analysis of the proximal 5′-untranslated region of ABCB gene from two mussel species revealed several putative binding sites for PKA-regulated TFs, which also occur within the promoter of the human ABCB1 gene [28]. Although specific studies on TF binding activities and ABCB promoter function in mussels are not available, these findings do support the hypothesis that activity of the mussel ABCB promoter is linked to the cAMP/PKA pathway. Furthermore, the identification of heat shock elements (HSE) in the promoter region of mussel ABCB gene provides structural evidence that the ABCB transcript is co-expressed with HSP70 gene products in response to elevated temperatures or further stressors triggering HSF transactivation [49]–[52].

In conclusion, the current study provides the first clear evidence for the cAMP/PKA-mediated regulation of the *ABCB* mRNA expression in mussels (**Fig. 8**). This is a significant finding given the crucial role displayed by the Pgp transporter encoded by the ABCB gene in the response of mussels to environmental stressors and their adaptation to these stressors.

Various studies reported that cAMP levels are increased by metals or other environmental contaminants [9], [53], [54], therefore the induction of cAMP-dependent *ABCB* transcription may serve as a defense strategy. Nevertheless, this study also demonstrated that *ABCB* transcription may be negatively modulated by at least an endogenous regulator, i.e. 5-HT, through a highly evolutionally conserved pathway. Therefore, pollutants able to interact with the 5-HT pathway have the potential to negatively affect the cAMP-signaling pathway and impair the ability of mussels to cope with environmental stressors, thus potentially mining their fitness.

## Materials and Methods

### Materials

The DetectX™ direct cyclic AMP enzyme immunoassay kit was purchased from Arbor Assays (US). The PepTag PKA assay kit was from Promega (Milan, Italy). Random primers and the RevertAid MulV reverse transcriptase were from Fermentas (Milan, Italy). The ChargeSwitch total RNA cell kit and the Fast Sybr Green reaction mix were from Life Technologies (Milan, Italy). Fluoxetine (FX), propranolol (PROP), noradrenaline (NOR), serotonin (5-HT), forskolin (FSK), dibutyryl cAMP (dbcAMP), H89 (N-[2-((p-Bromocinnamyl)amino)ethyl]-5-isoquinolinesulfonamide dihydrochloride), protease inhibitor cocktail (P8340), and all other reagents were from Sigma Aldrich (Milan, Italy).

### Mussel handling

Mytilus galloprovinicialis (4 to 6 cm in length) were obtained from a government certified mussel farm (Cooperativa Copr.al.mo, Cesenatico, Italy). They were transferred to the laboratory in seawater tanks and acclimated for 3 days in aquaria containing 35-psu filtered seawater at 16°C with continuous aeration (>90% oxygen saturation). Mussels were fed once a day with a commercial algal slurry (Koral, Xaqua).

### *In vivo* exposure experiments

Thirty mussels per treatment (6 individuals per vessel) were exposed for 7 days to 0.3 ng/L PROP, to 0.3 ng/L FX, or to the mixture FX+PROP (0.3 ng/L+0.3 ng/L). A group of unexposed (control) mussels were maintained in parallel to the treatment groups. Seawater was renewed each day and the chemicals added as stock solutions prepared in distilled water. Mussels were fed once a day. At the end of the exposure period, haemolymph was extracted from the posterior adductor muscle of each mussel using a sterile 1-mL syringe, centrifuged at 800×*g* for 10 min, snap frozen in liquid nitrogen and stored at −80°C.

### Haemocyte preparation and *in vitro* experiments

Hemolymph was extracted from the posterior adductor muscle of a number of individuals using a sterile 1-mL syringe then pooled to obtain the total volume required for each experiment. Hemolymph was then plated in 12-well plates (1 mL/well), and haemocytes were allowed settled for 1 h at 16°C in the dark. Cell attachment to the bottom of the well was checked microscopically. The medium was then removed, and cells were washed twice with 35-psu sterile artificial seawater (ASW). Control cells were incubated with 1 mL ASW, whereas 1 mL ASW containing the tested chemicals at the selected concentrations was added to the experimental wells. FSK and H89 were added to ASW from concentrated stock solutions prepared in dimethylsulphoxide (DMSO). In all cases, DMSO final concentration was 0.01% v/v and it did not significantly affect the biological endpoints analysed (data not shown). Experimental conditions (time of incubation and agonist/modulator concentrations) were assessed in preliminary trials to ensure significant evaluations of mRNA expressions along with cell signaling mediators. All incubations were carried out at 16°C in the dark. Each treatment consisted of 3 independent experiments, and each experimental trial consisted of 3 replicates for each biological endpoint (N = 3).

### cAMP assay

Haemocytes were lysed in 65% ethanol for 1 h at 4°C. Lysates were centrifuged (2000×g for 15 min at 4°C), and the collected supernatants were dried under a stream of nitrogen. Contents of cAMP were assessed using the DetectX™ direct cyclic AMP enzyme immunoassay kit according to the manufacture's protocol. Total protein content was assessed using the Lowry method [55]. cAMP levels were expressed as pmol/mg protein.

### PKA activity assay

Haemocytes were lysed in cold PKA extraction buffer containing 25 mM Tris-HCl pH 7.4, 0.5 mM EDTA, 0.5 mM EGTA, 10 mM β-mercaptoethanol and 50-fold diluted proteinase inhibitor cocktail. Supernatants were assayed for PKA activity using the non-radioactive PepTag PKA assay kit with dye-labeled Kemptide as a substrate according to manufacturer's protocol. Results were normalized to the total protein content and expressed as fold change vs control.

### Quantitative Real-Time PCR Analysis of *ABCB* and *5-HT_1_* mRNA Expressions

Total RNA was extracted from control and treated haemocytes using the ChargeSwitch total RNA cell kit according to the manufacturer's protocol. RNA concentration and quality were verified by UV spectroscopy and electrophoresis using a 1.2% agarose gel under denaturing conditions. First strand cDNA for each sample was synthesized from 1 µg total RNA in the presence of 250 ng random primers and 200 units RevertAid MulV reverse transcriptase following the manufacture's protocol.

Real-time PCR reactions were performed in duplicate, in a final volume of 10 µL containing 5 µL Fast Sybr Green reaction mix with ROX, 2 µL diluted cDNA, and 0.2 µM specific primers (Table 1). A control lacking cDNA template was included in the real-time PCR analysis to determine the specificity of target cDNA amplification. Amplification was detected with a StepOne real time PCR system apparatus (Life Technologies, Milan, Italy) using a standard “fast mode” thermal protocol. For each target mRNA, melting curves, gel pictures and sequences were analysed in order to verify the specificity of the amplified products and the absence of artifacts. The amplification efficiency of each primer pair was calculated using a dilution series of cDNA (Table 1). A normalization factor, calculated using the geNorm software [56] and based on the expression levels of the best performing reference transcripts in the haemocyte samples, was used for accurate normalization of real-time PCR data. A set of suitable reference genes were selected from the literature [57]–[60] and are listed in Table 1; amongst these, the most stable reference genes used for normalization in haemocytes subjected to the different treatments were 18S rRNA and EF-α1 (Table 1).

**Table 1:** List of primers used in real time PCR analyses.

Target/Reference mRNA	Primer sequence 5′-3′	Amplicon size (bp)	Amplification efficiency (%)	Reference
*MgABCB*	CACCATAGCCGAGAACATCC	139	112	This study*
	CTCCACGCTCTCCAACTAG			
*5-HT_1_*	CAGCTGCAAGATCGAGGATT	130	117	[41], [42]
	TGAAGCCATCTTGACTGACG			
*Actin*	GTGTGATGTCATATCCGTAAGGA	120	114	[58]
	GCTTGGAGCAAGTGCTGTGA			
*Tubulin*	TTGCAACCATCAAGACCAAG	135	102	[60]
	TGCAGACGGCTCTCTGT			
*Elongation factor α1*	CGTTTTGCTGTCCGAGACATG	135	99	[59]
	CCACGCCTCACATCATTTCTTG			
*RNA helicase*	GCACTCATCAGAAGAAGGTGGC	129	132	[60]
	GCTCTCACTTGTGAAGGGTGAC			
*18S*	TCGATGGTACGTGATATGCC	90	95	[57]
	CGTTTCTCATGCTCCCTCTC			
*28S*	AGCCACTGCTTGCAGTTCTC	142	94	[59]
	ACTCGCGCACATGTTAGACTC			

* primers were constructed basing on a partial sequence encoding an ABCB gene product from *M. galloprovincialis* (GenBank Ac Numb EF057747; [50]). A WWW-based database search using the BLAST program at NCBI found that this partial sequence showed a 65.17% and 64.93% nucleotide sequence identity with the human ABCB1 (GenBank Ac. Numb. NM_000927) and ABCB4 (GenBank Ac. Numb. NM_01884) gene sequences, respectively, while sequence identities lower or close to 50% were found with other human ABCB subtypes. Therefore, to avoid misleading information, the Pgp encoding gene from mussel investigated in this study will be referred to as ABCB gene.

Relative expression of target genes in comparison with those of the reference genes was calculated by a comparative C_t_ method [61] using the StepOne software tool (Life Technologies, Milan, Italy). Data were finally reported as normalized relative expression (fold change) with respect to control samples.

### Computational analysis of the promoter region of ABCB genes from mussels

Two full length sequences encoding ABCB genes from Mytilus californianus (GenBank Ac. Numb. EF52141) and M. galloprovincialis (GenBank Ac. Numb. FM999809) were retrieved from the GenBank database. The ORF Finder Tool at the NCBI (http://www.ncbi.nlm.nih.gov/gorf/gorf.html) was used to identify the putative 5′-untraslated regions of both sequences. Sequence of the human ABCB1 promoter in the same region is reported for comparison (GenBank Ac. Numb. NM000927). Regulatory elements in the 5′-untranlasted regions were identified with Alibaba2 [62], MATCHTM [63] and the Transcription Element Search System (TESS) [64].

### Statistical analysis

Real time PCR data were evaluated with the REST software [65] that uses a randomisation test with a pairwise reallocation to assess the statistical significance of the differences in expression between each treatment-exposure group and the control. cAMP levels and PKA activities were analysed using the SigmaStat statistical package. Significant differences between samples were determined using one-way ANOVA followed by the multiple comparison Bonferroni's test. In any case, statistical difference was accepted when p<0.05.
